# Prevalence and clinical correlates of misophonia symptoms in the general population of Germany

**DOI:** 10.3389/fpsyt.2022.1012424

**Published:** 2022-11-21

**Authors:** Ewgeni Jakubovski, Astrid Müller, Hanna Kley, Martina de Zwaan, Kirsten Müller-Vahl

**Affiliations:** ^1^Department of Psychiatry, Social Psychiatry and Psychotherapy, Hannover Medical School, Hanover, Germany; ^2^Department of Psychosomatic Medicine and Psychotherapy, Hannover Medical School, Hanover, Germany; ^3^Department of Psychology, Psychotherapy Outpatient Clinic of Bielefeld University, Bielefeld, Germany; ^4^Department of Psychology, Clinical Psychology and Psychotherapy, University of Bremen, Bremen, Germany

**Keywords:** misophonia, autonomous sensory meridian response (ASMR), decreased sound tolerance, epidemiology, prevalence

## Abstract

**Introduction:**

Misophonia refers to a phenomenon in which affected individuals have a selective intolerance to sounds of mostly oral or nasal origin. This intolerance is typically associated with strong emotional reactions such as anger, irritation, and disgust. The aim of this study was to conduct the first large epidemiological survey to determine the prevalence of misophonia symptoms in the adult population in Germany.

**Methods:**

We conducted a large-scale representative population survey between December 2020 and March 2021. For this purpose, a sample of 2,519 people were visited in their households and assessed with the Misophonia Questionnaire (MQ) and the Amsterdam Misophonia Questionnaire (AMISOS-R) to document misophonic symptoms. The primary estimate of clinical misophonia symptoms prevalence was based on the MQ Severity Scale and a secondary estimate was based on the AMISOS-R. The survey further included self-ratings to measure perfectionism, not-just-right experience (NJRE), autonomous sensory meridian response (ASMR) and general health as well as demographic data.

**Results:**

Five percent of the sample scored equal or above the MQ Severity Scale threshold for clinical misophonia symptoms (5.9% based on AMISOS-R). Individuals with clinical misophonia symptoms had a higher rate of perfectionism, a higher occurrence of NJRE, higher susceptibility to ASMR, and a worse general health status than those scoring below the cut-off-score. All those factors also independently predicted the severity of misophonia symptoms in a multiple regression model.

**Conclusion:**

Misophonia is a frequent condition and should further be examined as an independent diagnostic entity.

## Introduction

Misophonia is described as a phenomenon of selective sound sensitivity to specific patterns of sounds (in particular eating and breathing sounds by other humans), which is paired with an intense aversive emotional and physiological reaction to those stimuli (1). Although a misophonia diagnosis does not exist in the diagnostic manuals yet, a consensus definition among researchers was reached this year (2). To this day, information on its prevalence and associated factors in the general population is very limited (2). At first, smaller studies were published on the prevalence rates of misophonic symptoms in predefined samples such as students, hospital staff, and psychiatric inpatients revealing clinically relevant symptoms of misophonia in 10%−20% of individuals (3–6). In 2021, a first prevalence study in the general population was published by Kiliç et al. (7). This study was conducted in Ankara City in Turkey and included 256 households with 541 individuals older 15 years of age. All households were visited by trained interviewers, who used a semi-structured clinical interview, which was specifically developed for that study, to diagnose misophonia. Based on this interview, the diagnosis of misophonia was made in 12.8% of participants of the study sample, while 78.9% reported being distressed by at least one out of 50 misophonic sounds. A more recent population survey from the UK reported a prevalence of 18% of bothersome misophonia symptoms in the general population (8). Further larger trials reporting prevalence data on misophonia symptoms include an online survey conducted in the U.S. by Guetta et al. (9) which found a rate of clinical misophonia symptoms of 13.5% in a sample of 297 healthy adults and a study from the UK by Naylor et al. enrolling 336 medical students reporting clinically significant misophonic symptoms in 49.1% of the sample population (3). Thus, from recent studies it is suggested that selective sound intolerance is very common and a high number of individuals seems to be affected by the phenomenon of misophonia.

Our study aimed to deepen the knowledge on the prevalence of misophonia in the general population by reporting data from a nation-wide, representative survey carried out in Germany in over 2,500 households. The primary goal was to establish the prevalence of clinically significant misophonia symptoms in the general population in Germany. The secondary goal was to examine the associations of clinically significant misophonia to perfectionism, autonomous sensory meridian response (ASMR), not-just-right experience (NJRE), and general health. The inclusion of these variables was based on associations reported in previous studies (10, 11). Based on results of two large studies (10, 11), we expected to see a higher proportion of perfectionism among people with misophonia as compared to unaffected individuals, more frequent ASMR experience, and a lower general health status.

## Materials and methods

### Data sampling and procedure

Data were collected between December 2020 and March 2021. The demographic consulting company USUMA (Berlin, Germany) works in cooperation with the University of Leipzig and carried out our survey.

The randomized sampling procedure was carried out in three steps: in the first step, Germany was divided into 53,000 areas containing from 350 to 700 households. Those areas were subjected to a random allocation procedure. In the second step, for each sampling area, 23 households were selected along a random route starting from a predefined starting address. In the final step, one target member was selected from each household *via* the Kish grid method (12) – a procedure that allows each family member to be randomly selected with the same likelihood. The individual needed to be at least age 16 and able to read and understand German language.

### Procedure of the assessment

All information and assessments were administered during one in-person visit by an experienced staff member at the subject's home. At first, all selected individuals were informed about the purpose of the study, about their right to withdraw consent at any time during as well as after the assessment and that their already-collected data would be deleted in that case. The assessment included an inquiry of sociodemographic data and a battery of paper and pencil self-assessment questionnaires.

### Assessment instruments

To assess a broader range of misophonia symptoms, we included two instruments used in previous studies: the Misophonia Questionnaire (MQ) and the Amsterdam Misophonia Scale-Revised (AMISOS-R). The MQ was developed by Wu et al. (5), for the assessment of misophonia in an undergraduate student population. The MQ consists of three subscales: (i) a Symptom Scale, (ii) an Emotions and Behaviors Scale, and (iii) a Severity Scale. The MQ Symptom Scale inquires about seven areas of sound sensitivity (such as eating, tapping, and nasal sounds), each rated on a scale from 0 (not at all true) to 4 (always true); (ii) the MQ Emotions and Behaviors Scale inquires about 10 emotional and behavioral reactions to misophonia triggers (such as getting angry or leaving the room) rated on the same 0 to 4 scale. Both scales are summed resulting in a MQ Total score which ranges from 0 to 68 (higher scores indicate a higher level of misophonia); and (iii) the MQ Severity Scale is a single-item-scale that instructs the respondent to rate their sound sensitivity on a scale from 1 “minimal” to 15 “very severe.” The MQ scales showed a good internal consistency in previous studies α = 0.86–0.90 (5, 6).

The AMISOS-R is a revised version of the Amsterdam Misophonia Scale published in 2013 by Schröder et al. (13). It consists of a brief symptom checklist, which is not included in the rating and a severity/impairment scale of 10 items (e.g., “To what extent do you experience impairment due to these sounds?”) of which each is rated from 0 (none) to 4 (extreme). The total score ranges from 0 to 40 (higher scores indicate a higher level of misophonia). The AMISO-R was shown to have a good internal consistency α = 0.84 (14). Both questionnaires were back to back translated into German by bilingual and professional translators and approved by the original authors (15, 16).

To examine possible clinical correlates of misophonia, we assessed perfectionism, NJRE, ASMR, and general health. We used a shortened, 8-item version of the Frost Multidimensional Perfectionism Scale (FMPS), which was proposed by Burgess et al. (17) and was shown to have good psychometric qualities. Items are scaled from 1 (strong disagreement) to 7 (strong agreement); summing to a total score ranging from 8 to 56.

The NJRE-Questionnaire Revised (NJRE-QR) is a 19-item self-report questionnaire to assess NJRE (18). NJRE describes an experience of something not being or feeling the way it is supposed to feel (18). The first 10 items are screening questions and the subsequent nine items are severity ratings for scaled from 1 (not intense at all) to 7 (extremely intense) resulting in a sum score ranging from 9 to 63.

The Short-Form Health Survey (SF-8) is an instrument for assessing health-related quality of life (HRQoL) (19). It consists of eight items, five of which are scored from 1 (good health) to 5 (bad health), for and two of which are score 1–6. The sum score ranges from 8 to 37 with lower scores indicating higher HRQoL.

Since, so far, there has been no instrument available for the assessment of ASMR, we included a screening question similar to that used by Rouw et al. (11) in their online survey to assess ASMR in people with misophonia: “Have you ever responded to whispering or to the sound of rubbing fingers against a rough surface in the following way: a pleasant tingling sensation on your head, scalp, back, or other parts of your body?” (yes/no).

### Determining individuals with clinical misophonia symptoms

Misophonia Questionnaire Severity Scale assesses the patient's impairment directly and is a good candidate for a cut-off for a diagnostic cut-off. In accordance with previous studies (5, 6), we used a cut-off score of >7 on the MQ Severity Scale to diagnose clinically significant misophonia symptoms. In addition, previous studies utilized the MQ Symptom Scale at a cut-off of >14 as a screening to determine the amount of people who are “sometimes” sensitive to certain sounds on average (5, 6).

In addition, a secondary criterion for clinical misophonia symptoms was established, using the AMISOS-R. For the AMISOS-R no cut-off value is provided. Based on data from the currently largest study with a sample of 575 subjects with misophonia by Jager et al. (10), we calculated a cut-off value by using one SD (6.46) below the reported mean AMISOS-R score (29.78) as the cut-off (>23) (10).

### Statistical analysis

All statistical analyses were conducted with SPSS statistical package version 26. Cronbach's alpha was calculated for MQ total score and AMISOS-R total score. Depending on the analysis, we divided the sample into two subgroups with and without clinical misophonia symptoms using the cut-off of 7 on the MQ Severity Scale and the score of (above) 23 for the AMISOS-R. We computed frequencies, percentage, means, and SD where appropriate. For statistical comparison Chi-square tests or independent sample *t*-tests were computed. For group comparisons Cohen's *d* was computed as a measure of effect size. For correlation analysis the Spearman correlation coefficient was used. Three multiple linear regression models were used to examine the predictive values of the clinical variables assessed in this study. The predictor variables included age and gender as control variables, FMPS, NJRE-QR, SF-8, and ASMR. Apart from ASMR, which was a binary variable (present/absent), all other predictors were continuous. We calculated prediction models for three different dependent variables to cover broader aspects of misophonia: MQ Total Score, MQ Severity Score, and the AMISOS-R total score. All statistical comparisons were tested on significance level of *p* < 0.05.

### Ethics statement

This national-wide survey was carried out in accordance with the ethical guidelines of the International Code of Marketing and Social Research Practice by the International Chamber of Commerce and the European Society for Opinion and Marketing Research. The ethics committee of the University of Leipzig reviewed and approved the study.

## Results

### Sampling and assessment procedure

**Figure 1:**
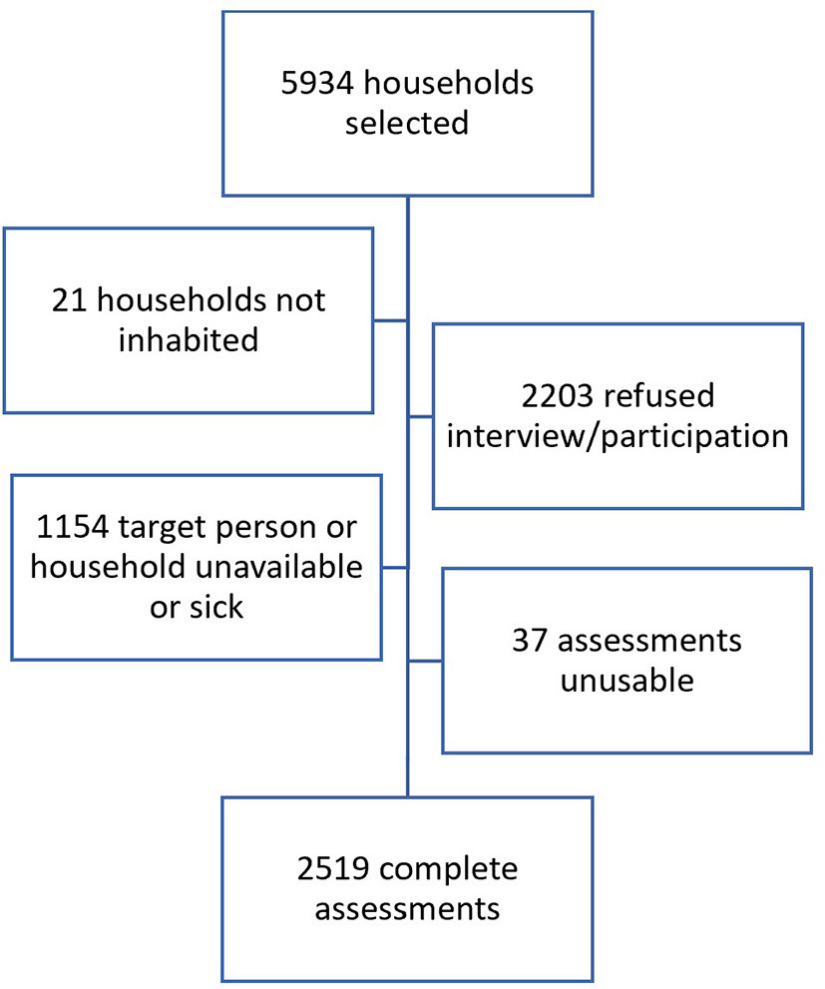
Flowchart recruitment of participants.

A total of 5,934 selected households were selected, out of which, 21 were not inhabited, 1,154 were not available for an assessment or sick, and 2,203 were unwilling to participate. A total of 2,556 assessments were made, out of which 37 were not valid, resulting in a total of 2,519 complete assessments (Figure 1).

The misophonia symptom scales were tested for internal consistency. Cronbach's alpha was 0.90 for MQ total and 0.89 for AMISOS-R. Both scales correlated in the medium high range at *r* = 0.72 (*p* < 0.01). Correlations with other assessments in terms of divergent validity ranged from −0.01 until 0.37 for AMISOS-R total score and from −0.12 until 0.40 for MQ total score (for details see Table 3).

### Sample characteristics

To illustrate the representativeness of our sampling procedure (Table 1) compares our sample to the data from the German census bureau for the year 2019. Sample characteristics are displayed in Table 2. Out of the total of 2,519 participants, 1,322 (52.5%) were female and 1,197 (47.5%) were male. The average age was 50.3 years (SD: 18.1; range: 16–96 years). Further, 1,141 (45.5%) were married, 1,946 (77.9%) had a high school degree, and 152 (6.0%) were unemployed.

**Table 1:** Comparison of our sample with the General Population in Germany.

**Variable**	**Our sample (%)**	**General population (%)**
**Size of household**		
1 person	36	41.9
2 persons	38	33.8
3 persons	13.7	11.9
4 persons or more	12.3	12.4
**Age distribution men**		
16–19	1.6	2.4
20–29	5.8	7.1
30–39	7.3	7.6
40–49	7.2	7.3
50–59	9.7	9.4
60–69	8.8	7
70+	6.9	7.7
**Age distribution women**		
16–19	1.9	3.2
20–29	6.5	6.5
30–39	7.7	7.3
40–49	8.7	7.2
50–59	10.2	9.4
60–69	8	7.4
70+	9.6	10.5
**German states**		
Schleswig-Holstein	3.9	3.5
Hamburg	2.4	2.2
Lower Saxony	9.6	9.6
Bremen	0.8	0.8
Northrhine-Westphalia	21.3	21.5
Hesse	6.6	7.5
Rhineland Palatinate	4.4	4.9
Baden-Württemberg	11.7	13.3
Bavaria	14.3	15.7
Saarland	1	1.2
Berlin (West)	3.5	2.4
Brandenburg	3.8	3
Mecklenburg Western Pomerania	2.4	2
Saxony	6.6	4.9
Saxony-Anhalt	2.5	2.7
Thuringia	3	2.6
Berlin (East)	2.3	2

Data originated from German census bureau for 2019.

**Table 2:** Demographic information on the whole sample and subgroups (with and without clinical misophonia symptoms).

	**Total**		**No misophonia**		**Misophonia**	
Gender, female, *n*, %	1,322	52.5%	1,250	52.2%	72	57.1%
Age, mean, SD	50.3	18.06	50.2	18.09	52	17.33
**Age groups, years**, ***n*****, %**						
16–24	229	9.1%	222	9.3%	7	5.6%
25–34	364	14.5%	345	14.4%	19	15.1%
35–44	399	15.8%	376	15.7%	23	18.3%
45–54	411	16.3%	395	16.5%	16	12.7%
55–64	488	19.4%	464	19.4%	24	19.0%
65–74	390	15.5%	365	15.3%	25	19.8%
>75	238	9.4%	226	9.4%	12	9.5%
Marital status, married, *n*, %	1,141	45.5%	1,081	45.3%	60	47.7%
Part of Germany (west vs. east), *n*, %	2,000	79.4%	1,899	79.4%	101	80.2%
Rural vs. urban, *n*,%	309	12.3%	286^*^	12.0%^*^	23^*^	18.3%^*^
Degree, High school (Abitur), *n*, %	1,946	77.9%	1,838^*^	77.5%^*^	108^*^	86.4%^*^
Unemployed, *n*, %	152	6.0%	147	6.1%	5	4.0%
Experience of ASMR, *n*, %	1,013	41.7%	950^*^	41.2%^*^	63^*^	50.4%^*^

Clinical Misophonia Symptoms established via MQ Severity Scale >7.

* Group difference significant on a p > 0.05 level.

### Prevalence estimate of clinical misophonia symptoms

In our sample, 126 individuals, which corresponds to 5.0% of the total sample, had a MQ Severity of >7 and were considered as suffering from clinical misophonia symptoms. As far as trigger sensitivity was concerned, 81 participants (3.2%) had an MQ Symptom scale score of >14. The secondary cut-off criterion of an AMISOS-R rating of >23 was met for 148 individuals, which corresponded to 5.9% of the total sample. The agreement *via* kappa was at 0.37, which is considered fair (20). Fifty-five individuals (2.2%) fulfilled both cut-off criterions.

Individuals with clinical misophonia symptoms did not differ from individuals without in most demographic variables including age and gender. However, individuals with clinical misophonia symptoms had a higher rate of high school degrees [86.4 vs. 77.5%; OR: 1.66 (CI: 0.99–2.77), *p* < 0.05], lived in rural areas [18.3 vs. 12.0%; OR 1.60 (Cl: 1.03–1.60), *p* < 0.5] and reported more frequently ASMR experiences [50.0 vs. 41.2%; OR: 1.45 (CI: 1.01–2.08), *p* < 0.05; Table 2].

### Correlates of misophonia

Table 3 displays the Spearman correlations of all clinical scales used in this survey. Apart from correlation between MQ subscales, a medium to large correlation was seen between AMISOS-R and MQ Total. Both misophonia scales had a medium to low positive correlations with NJRE-QR (AMISOS-R: *r* = 0.37; MQ: *r* = 0.40, *p* < 0.01) and SF-8 (AMISOS-R: *r* = 0.37; MQ: *r* = 0.31, *p* < 0.01), and a low positive correlation with FMPS (AMISOS-R: *r* = 0.22; MQ: *r* = 0.15, *p* < 0.01). AMISOS-R did not correlate with the ASMR item, while MQ had a low negative correlation (*r* = −0.12, *p* < 0.01). Age did not correlate with AMISOS-R or MQ total, however, it showed a significant low positive correlation with the MQ Severity Scale of *r* = 0.04 (*p* < 0.05).

**Table 3:** Spearman correlations among misophonia scales and other clinical scales.

	**AMISOS-R total score**	**MQ total score**	**MQ Severity Scale**	**MQ Symptom Scale**	**MQ Emotions and Behavior Scale**	**FMPS 8**	**ASMR**	**NJRE-QR**	**SF 8**
AMISOS-R total score	1.000	0.715^**^	0.590^**^	0.546^**^	0.713^**^	0.220^**^	−0.010	0.365^**^	0.369^**^
MQ total score		1.000	0.651^**^	0.891^**^	0.883^**^	0.146^**^	−0.124^**^	0.404^**^	0.314^**^
MQ Severity Scale			1.000	0.727^**^	0.605^**^	0.098^**^	−0.169^**^	0.271^**^	0.211^**^
MQ Symptom Scale				1.000	0.610^**^	0.085^**^	−0.190^**^	0.251^**^	0.236^**^
MQ Emotions and Behavior Scale					1.000	0.204^**^	−0.089^**^	0.396^**^	0.292^**^
FMPS 8						1.000	−0.124^**^	0.253^**^	−0.001
ASMR							1.000	−0.201^**^	−0.026
NJRE-QR								1.000	0.417^**^
SF-8									1.000

AMISOS-R, Amsterdam Misophonia Scale-Revised; MQ, Misophonia Questionnaire; FMPS, Frost Multidimensional Perfectionism Scale; ASMR, autonomous sensory meridian response; NJRE-QR, Not Just Right Experience-Questionnaire Revised; SF-8, Short Form Health Survey.

** Significant on a *p* < 0.01 level.

### Group comparisons

Table 4 displays averages and SDs on clinical assessment overall and by diagnostic groups (misophonia yes/no). As expected, people with clinical misophonia symptoms had significantly and markedly higher values on both the MQ total and the AMISOS-R total. Apart from that, individuals with clinical misophonia symptoms had higher values on the FMPS (20.8 vs. 18.1, Cohen's *d* = 0.39), the NJRE-QR (20.8 vs. 16.2, Cohen's *d* = 0.59), and the SF-8 (19.9 vs. 14.2, Cohen's *d* = 0.77).

**Table 4:** Group comparison of clinical variables.

	**Total**		**No Misophonia**		**Misophonia**		**Effect size**
	**Mean**	**SD**	**Mean**	**SD**	**Mean**	**SD**	**Cohen's *d***
FMPS	18.23	7.01	18.10	6.97	20.82	7.24	0.39
NJRE-QR	16.60	7.68	16.21	7.51	20.76	8.27	0.59
SF-8	15.06	6.62	14.8	6.47	19.91	7.48	0.77
MQ total score	8.91	8.65	7.70	7.43	21.09	10.57	1.55
MQ Symptom Scale	2.51	4.13	2.14	3.67	9.39	5.97	1.76
MQ Emotions and Behavior Scale	4.15	5.09	3.47	4.4	11.18	6.27	1.51
MQ Severity Scale	1.53	2.58	1.11	1.77	9.48	2.70	3.24
AMISOS-R total score	18.61	6.50	17.51	5.71	25.65	6.85	1.25

AMISOS-R, Amsterdam Misophonia Scale-Revised; MQ, Misophonia Questionnaire; FMPS, Frost Multidimensional Perfectionism Scale; NJRE-QR, Not Just Right Experience-Questionnaire Revised; SF-8, Short Form Health Survey.

All group comparisons are significant on a *p* < 0.001 level.

### Regression analysis

Three multiple linear regressions models were calculated. The prediction models were computed for the MQ total score, the MQ Severity Scale, and the AMISOS-R total score as dependent variables. The results are displayed on Table 5. In all prediction models, the same predictors were entered. The variables age and gender were entered as control variables and were not significant in any model. For the MQ total score and the MQ Severity Scale, NRJE-QR was the best predicting variable with the highest standardized beta (MQ Total: β = 0.32, *p* < 0.01; MQ Severity: β = 0.15, *p* < 0.01), while for the AMISOS-R total score SF-8 was the best predictor (β = 0.264, *p* < 0.01). The other discrepant result among the models refers to the FMPS which was only significant in the MQ severity model (β = 0.10, *p* < 0.01). As far as the amount of explained variance was concerned, the *r*-square was 0.27 for the MQ total score, 0.10 for the MQ Severity Scale, and 0.18 for the AMISOS-R total score.

**Table 5:** Predictors of MQ total score and MQ Severity in multiple linear regression models.

**Variable**	**Model 1: MQ total score**			**Model 2: MQ Severity**			**Model 3: AMISOS-R total score**		
	**Standardized beta**	***t***	***p*-Values**	**Standardized beta**	***t***	***p*-Values**	**Standardized beta**	***t***	***p*-Values**
Gender	0.030	0.818	0.414	0.021	0.678	0.498	0.021	0.410	0.682
Age	−0.023	−0.578	0.563	0.043	1.260	0.208	−0.081	−1.469	0.143
FMPS 8	0.073	1.877	0.061	0.100	3.017	0.003	−0.017	−0.315	0.753
NJRE-QR	0.324	7.602	0.000	0.151	4.136	0.000	0.221	3.673	0.000
SF 8	0.180	4.150	0.000	0.123	3.267	0.001	0.264	4.353	0.000
ASMR	−0.165	−4.472	0.000	−0.084	−2.633	0.009	−0.070	−1.341	0.181
R-Square	0.272			0.096			0.182		

MQ, Misophonia Questionnaire; FMPS, Frost Multidimensional Perfectionism Scale; NJRE-QR, Not Just Right Experience-Questionnaire Revised; SF-8, Short Form Health Survey; ASMR, autonomous sensory meridian response; AMISOS-R, Amsterdam Misophonia Scale-Revised.

## Discussion

We report the data from the first large representative German population survey on misophonia including a total of 2,519 individuals. Our results indicate that 5% of the general population suffers from clinical misophonia symptoms according to the recommended cut-off score of the MQ Severity Scale. Our secondary cut-off using the AMISOS-R scale indicated a prevalence of 5.9%. In addition, individuals with misophonia - as opposed to unaffected individuals - have higher perfectionism, higher occurrence of NJRE, higher susceptibility to ASMR, and a worse general health status. All those factors also independently predicted the severity of misophonia symptoms in a multiple regression model.

The AMISOS-R had a higher correlation with MQ total score, which is a sum of the Symptom and the Behavior Scales of the MQ, than with the MQ Severity Scale, suggesting a greater conceptual closeness. This also explains the relative low kappa between the two different cut-offs. Although both criteria identified a similar amount of people affected by misophonia the identified individuals were not always identical. Since the MQ Total Score cut-off was more established we sticked with that criterion. The AMISOS-R correlated significantly with perfectionism, NJRE and SF-8, which corroborated the results of the MQ scales. In the regression model it was only significantly predicted by NJRE and SF-8, showing no effect of perfectionism and ASMR.

Somewhat surprisingly, the prevalence found in our study is lower than in all the studies published so far (3, 5–7, 21). Remarkably, Kiliç et al. (7) found a prevalence more than twice as high compared to our study, although they used a quite rigorous interview procedure to establish a diagnosis of misophonia instead of a self-assessment only as we did. Moreover, Guetta et al. (9) who investigated misophonia in an online sample of 297 healthy adults from the U.S. using the MQ, found a rate of clinical misophonia symptoms of 13.5%, which was very similar to the study by Kilic et al. (7). Therefore, the low prevalence found in our study cannot be explained by our use of the MQ alone. One might speculate, that the prevalence of clinical misophonia symptoms in Germany is lower than that in Turkey or the U.S. Alternatively, differences might be explained by the different methodological approaches: while our study is not only the first one in Germany and the largest, it is also the most representative study conducted so far, since a representative cross-section of the population was included from all areas of a country, i.e., rural and urban areas, while Kiliç et al.'s (7) study only took place in Ankara. Interestingly, in our sample individuals with clinical misophonia symptoms were more likely to be found in rural areas. Possibly individuals suffering from misophonia prefer rural areas over urban areas because of the lesser amounts of triggers on the country side. Although it does not explain the higher prevalence of misophonia in the Kiliç et al.'s study (7), our finding emphasized the importance for prevalence studies to include both urban and rural areas for a more precise prevalence estimate. The use of two different misophonia scales improved the validity of our results. We found AMISOS-R to have a higher correlation with the MQ total score than with the MQ Severity Scale. The MQ total score assesses the variety of symptoms while the MQ Severity Scale assesses the patient's impairment and suffering from misophonic symptoms. The AMISOS-R also corroborated the results of the MQ by showing similar significant correlations with perfectionism, NJRE, and SF-8.

While Kiliç et al. (7) found a predominance of females of about 70% with misophonia symptoms in their samples, we found no significant gender differences. In a similar vein no gender difference in the occurrence of misophonia was reported by Aryal and Prabhu (22), who examined misophonia in a sample of 172 students in India. As far as associations between gender and misophonia symptoms are concerned, we have also not found gender to be a predictor of misophonia symptoms similarly to Wu et al. (5), who reported no association between gender and misophonia symptoms as measured by the MQ total score (however, the severity scale was not examined). In contrast Vitoratou et al. (8) reported higher symptom severity in men as compared to women with clinical misophonia symptoms. While being noteworthy, we do not have any explanation for this discrepancy at the current time. Furthermore, Kiliç et al. (7) found younger age to be predictive of misophonia symptom severity and Rouw et al. (11) reported a negative correlation between age and symptom severity. However, when looking at the data more closely they discovered a trend that resembled more a curvilinear relationship with misophonia symptom severity decreasing in the 30, 40, and 50 s and increasing again thereafter. Our data did not indicate any particular age category to have a higher prevalence of misophonia, however, a small negative correlation could be established when age was correlated with the MQ Severity Scale. While age and gender were entered in all prediction models as control variables, no significant prediction effect could be established. In sum, our data do not support any significant role of gender or age in relationship to misophonia symptoms.

Perfectionism is a personality characteristic that was found to be associated with misophonia by a number of researchers. Schröder et al. (13), who first suggested diagnostic criteria for misophonia, developed their first rating scale in accordance with the Yale-Brown Obessive-Compulsive Scale (Y-BOCS) – the gold standard assessment for obsessive-compulsive disorder (OCD) and reported a comorbidity rate for obsessive-compulsive personality disorder (OCPD) of 52.4%. Increased rates of comorbid obsessive-compulsive behavior (5, 6) and OCD (up to 15.9%) (7) were reported by some studies. Jager et al. (10) in contrast reported 26% of their participants to have traits of OCPD, while the diagnosis of OCPD was given in only 2.4%. However, 97% were found to have clinical perfectionism. In the same vein, perfectionism was significantly higher in our participants with misophonia as compared to those without. In addition, we found perfectionism to have a weak positive correlation with misophonia total symptom scores and to be of predictive value for the MQ Severity Scale. Therefore, our study adds further evidence to the hypothesis that perfectionism is playing a role in misophonia.

Related to the phenomena of perfectionism and OCD are NJRE. Coles et al. (18) suggested that NJRE might represent a form of “sensory perfectionism”. Our study is the first to examine NJRE in relation to misophonia symptoms in an exploratory way. In accordance with our expectations, NJRE was more pronounced in individuals with misophonia. It showed the highest correlations with the AMISOS-R total score and the MQ total score and was shown to be the best predictor of misophonia symptoms as measures by the MQ total score and the MQ Severity Scale. Notably, in the regression models NJRE predicted misophonia symptoms independently of perfectionism. Thus, from our data it is suggested for the first time that NJRE and misophonia symptoms are closely related.

Autonomous sensory meridian response is a phenomenon that to date received little attention by the research community and research studies are scarce (23–26). To our knowledge, our study is the first population-wide survey to examine that phenomenon. We found self-reported ASMR susceptibility to be present in 41.7% of the sample. As expected, individuals with clinical misophonia symptoms had a higher ASMR susceptibility as compared to unaffected individuals. This corroborates the results by Rouw et al. (11) reporting ASMR in 50% of people with misophonia. At the same time, ASMR had a low negative correlation with the severity of misophonia symptoms as measured by the MQ and no significant correlation with the AMISOS-R, which contradicts the hypothesis that ASMR susceptibility is linearly connected to misophonia symptoms. One might hypothesize that ASMR susceptibility could be heightened in individuals who are generally susceptible to misophonia triggers, but that the pleasantness of the ASMR experience could decrease with greater symptom severity. However, it should be mentioned that ASMR was measured by one screening question only and that for more valid statements a dedicated study with broader assessments for both ASMR and misophonia symptoms would be necessary.

Several studies have established that individuals with misophonia have a lower quality of life and functioning in everyday life (5, 6, 14). In our study, we examined the health status of the participant using the SF-8 and - as expected – found a worse general health and functioning in the people with clinical misophonia symptoms.

Our study has few notable limitations, (i) methodologically this study was not conducted by trained mental health professionals and relied on people's own judgement when filling out the self-assessment questionnaires. With this limitation in mind, we chose scales that were already successfully used in large surveys, and for which comparable data exist. (ii) Due to the large sample size and associated high costs of the survey, we could only include the shortest scales in the survey, which made our assessment package relatively small. (iii) The regression models explained only a small amount of variance in misophonia severity. However, regression modeling was not a primary goal of our study and should be viewed as exploratory. (iv) The questionnaires we employed do not address visual triggers, which might capture a broader range of severity and impairment than auditory triggers alone. (v) Due to the brevity of the available survey space, we have not included an assessment of hyperacusis. Since some of the symptoms are similar among sound intolerance disorders we cannot exclude a degree of confounding with hyperacusis in our study. However, current studies suggest that the presents of hyperacusis in patients with misophonia is very low (10). That being said, our study's greatest strengths are its large sample, which constitutes the largest study on misophonia to date, and its representativeness, due to our highly sophisticated sample selection.

## Conclusion

Our study indicates that 5% of the general population in Germany are affected by clinically significant misophonia symptoms. Currently, however, no diagnosis of misophonia can be officially given to those who are affected. In addition, no evidence-based specialized treatment is available although individuals with misophonia have a lower quality of life and functioning in everyday life. Our study therefore emphasizes the need for diagnostic clarity and treatment options as well as for further study efforts in this new field to explore conceptual closeness and discrepancy to other (mental) disorders, and derive ideas for interventions that may reduce the burden of this sound intolerance in daily life.

## Data availability statement

The original contributions presented in the study are publicly available. This data can be found here: https://doi.org/10.17632/zjrt56tcnx.1.

## Ethics statement

The studies involving human participants were reviewed and approved by Ethics Committee of the University of Leipzig. Written informed consent to participate in this study was provided by the participants themselves in the case of adults or their legal guardian/next of kin in the case of minors.

## Author contributions

EJ designed the study, carried out the statistical analysis, and wrote the first draft. KM-V, MZ, and AM were involved in planning of the study. HK was involved in the translation of the questionnaires and consulted on the analysis plan. KM-V and MZ were involved the financial aspects of the survey. All authors edited the manuscript and agreed to the final version.

## Conflict of interest

The authors declare that the research was conducted in the absence of any commercial or financial relationships that could be construed as a potential conflict of interest.

## Publisher's note

All claims expressed in this article are solely those of the authors and do not necessarily represent those of their affiliated organizations, or those of the publisher, the editors and the reviewers. Any product that may be evaluated in this article, or claim that may be made by its manufacturer, is not guaranteed or endorsed by the publisher.
